# Population, demographic and socioeconomic characteristics associated with state preemption laws in the United States, 2009–2018

**DOI:** 10.1371/journal.pone.0321184

**Published:** 2025-04-04

**Authors:** José A. Pagán, Diana Silver, Kelley Akiya, Jennifer L. Pomeranz

**Affiliations:** Department of Public Health Policy and Management, New York University School of Global Public Health, New York, NY, United States of America; Islamic Azad University Urmia Branch, IRAN, ISLAMIC REPUBLIC OF

## Abstract

**Objective:**

In the United States, preemption laws enacted by state governments can remove local government authority to enact policy and undermine community self-determination and local democracy. No study to date has evaluated the population, demographic, and socioeconomic characteristics associated with state preemption of public health policies. Our study identifies state characteristics associated with preemption of local paid sick leave, food and nutrition, tobacco control, and firearm safety policies.

**Methods:**

We conducted a Classification and Regression Tree (CART) analysis using state-level demographic, socioeconomic, and population health indicators from 2009 to 2018 to predict state ceiling preemption of local paid sick leave, food and nutrition, tobacco control, and firearm safety policies.

**Results:**

Several demographic, economic, political, and health factors best distinguish states with and without preemption in each of the four domains. Total state population was an important characteristic in all four trees and the non-Hispanic Black population was important in three trees. All other age- and race/ethnicity-related demographic variables included were important characteristics in at least one tree. Additionally, adult obesity and flu vaccination were relevant in the paid sick leave tree and firearm-deaths, suicide-deaths, and the unemployment rate were relevant in the firearm safety tree. The relationship between specific factors and preemption in each of the four domains varied depending on the location of the factor within the trees.

**Conclusions and relevance:**

Specific population, demographic and economic characteristics in a state are associated with the adoption of ceiling preemption of paid sick, food and nutrition, tobacco, and firearm safety laws, but these characteristics vary by domain. Our study identified which populations within groups of states may be affected by preemption. The findings can inform whether preemption laws considered or adopted in a state may also require protective measures for population groups that could be adversely affected by these laws.

## Introduction

The United States is made up of fifty states that have political subdivisions (e.g., towns, counties, cities) that are referred to as local governments. States have enacted laws that preempt or remove or limit the authority of local governments to enact laws on a given issue; this is called “ceiling preemption” (hereinafter preemption) [1]. State preemption laws vary in their breadth but generally result in concentrating power in state legislatures while limiting the capacities of local governments–those closest to the people–to respond to the needs of their community members [2,3].

Although preemption may be a useful tool for state governments to achieve public health goals or create consistency in policymaking across different local jurisdictions, preemption is frequently passed without such state-level policy action [4–7]. This prevents localities from protecting their communities from harms that may not be apparent at the state level or may be a way to remove local policymaking authority in areas important to public health [1]. Indeed, state preemption is often passed at the behest of the industries that localities seek to regulate to protect the health and safety of their community members [8,9]. Thus, state preemption often functions as a form of deregulation for businesses while undermining community self-determination and local democracy [8].

This paper focuses on state preemption in four public health policy domains that have different histories and evidence-bases: tobacco control, firearm safety, paid sick leave, and food and nutrition policy. Many states preempted local control over tobacco in the 1980s and firearm safety in the 1990s [9,10], while states have preempted local control over paid sick leave laws and food and nutrition policy more recently [5,11]. There is strong evidence that tobacco control policies (e.g., taxes [12], indoor smokefree air acts [13]) are associated with decreased smoking and that mandatory paid sick leave is associated with lower risk of all-cause mortality and increased use of preventive and healthcare services [14,15]. In contrast, new and emerging evidence has demonstrated that specific firearm safety laws (e.g., lock box requirements) and food and nutrition policies (e.g., soda taxes, junk food taxes) are associated with decreased firearm-related violence [16,17] and healthier diets [18,19], respectively. When a state preempts the ability of local governments to enact public health policies, it removes an avenue for local elected officials to protect their community members through evidence-based policymaking or to experiment with solutions to public health problems on a local level [20].

This study identifies the population, demographic, and socioeconomic characteristics associated with state preemption of public health policies in the four policy domains. Understanding which state-level characteristics may be associated with preemption may be useful to policymakers, public health practitioners, and advocates interested in determining which states may be more likely to enact preemptive laws as well as identifying which populations could be affected by these laws. Using 2009-2018 data from all 50 states, we coded state statutes and conducted Classification and Regression Tree (CART) analysis to identify the different groups of states that have preemption laws related to the four domains. We selected the 2009-2018 ten-year timeframe because, while tobacco and firearm preemption laws have been in place for many years, state preemption of paid sick leave and food and nutrition policy increased after 2009.

## Materials and methods

We sourced state statutes that included ceiling preemption in the four domains from well-established non-profit organizations’ websites [21–27] which tracked preemptive laws for the years of the study: 2009-2018. We retrieved sourced statutes and all other statutes within the same code sections from Lexis + . Following established standards for coding legal statutes [28], we recorded the presence of ceiling preemption in each state and year. For statutes enacted prior to 2009, we established that they were still in effect and coded them accordingly. For statutes that became effective or were repealed during our ten-year timeframe, we recorded these dates and coded the year accordingly. The data used for our analysis is publicly available through the Inter-university Consortium for Political and Social Research data archive [29].

We retrieved data capturing the population, demographic, and socioeconomic characteristics of each state. Variables within these characteristics were selected because they capture factors that have been identified as associated with policy diffusion in the U.S. in prior studies examining public health policy diffusion and can be changed and/or monitored over time [30–33]. We obtained population and economic data from the American Community Survey, Current Population Survey, and U.S. Bureau of Economic Analysis including the population size; the percent of the population under age 18, ages 18 to 64, age 65 years and older; the percent identifying as Non-Hispanic (NH) White, NH Black, NH Asian and Pacific Islander (API), NH American Indian and Alaska Native (AIAN), and Hispanic; median income; per capita income; and the percent of the population in poverty, unemployed, and uninsured. Population health data included adult smoking and obesity prevalence from the Behavioral Risk Factor Surveillance System (BRFSS); flu vaccine coverage estimates from the Centers of Disease Control and Prevention (CDC); age-adjusted lung cancer deaths per 100,000 from the CDC’s US Cancer Statistics, and age-adjusted firearm-related deaths, homicide deaths, suicide deaths per 100,000 from the CDC’s National Vital Statistics System. Variables describing states’ political context included the percent of adults who voted (self-reported in the Current Population Survey), percent of eligible adults voting for the highest office (votes cast divided by the voting-eligible population estimated by the US Elections Project), state party control (Republican, Democrat, split), percent of the population receiving Temporary Assistance to Needy Families (TANF) calculated from the Office of Administration for Children and Families caseload data, and percent receiving Supplemental Nutrition Assistance Program (SNAP) benefits calculated from Food and Nutrition Service SNAP data tables. Data for all variables except those describing the voting population are collected annually. Voting data is available every two years and we used constant values for each two-year period for these variables. We obtained complete data for all years on all variables except for homicide deaths, which had missing data for seven percent of observations. All extracted data used for analysis is available in S1 File.

First, we calculated the number and the percentage of states with ceiling preemption laws in place during each year in the four policy domains (paid sick leave, food and nutrition policy, tobacco control, and firearm safety). We then used CART to identify which demographic, economic, political, or health factors were statistically important in explaining which states were more likely to have state preemption of paid sick leave, food and nutrition policy, tobacco control, and firearm safety from 2009 to 2018. The CART analysis was employed to predict whether a state had a preemption law in a given year but not whether it had the law prior to the year in question or whether it retained the law after the study period.

CART is a non-parametric statistical approach that uses binary recursive partitioning of data across multiple variables to identify distinct subgroups with a higher prevalence of a given outcome (in our case, preemption in one of four domains) [34]. Binary partitioning in CART facilitates the interpretation of results. The CART algorithm considers all explanatory variables (i.e., state characteristics) imputed to the analysis and all potential splits on each variable. In the case of binary variables (for example, Republican legislative control), a “split” is the presence or absence of the characteristic in a specific year and in the case of continuous variables, the split is a threshold value (for example, per capita income of $40,000). For continuous variables, the CART algorithm evaluates splits on every value of the variable. For example, in the case of per capita income, each dollar value is evaluated as a potential threshold. The algorithm then selects the split that partitions the data into two subgroups that are as different from one another as possible in terms of the outcome. When data is missing, the CART algorithm considers splits based on available non-missing values. This partitioning continues until no more significant splits are detected by the algorithm. Once the partitioning is complete, CART generates a tree consisting of branches representing each split and nodes representing the subgroups resulting from those splits. The algorithm allows us to describe which combination of demographic, economic, political, and health factors best distinguish states with and without preemption in a given year in each of the four domains.

Our dataset used for CART consisted of explanatory variables describing the states’ demographics, economy, political environment, and health needs during each year of our study (n = 500 state-years) and a binary preemption variable for each domain indicating whether the state had ceiling preemption in that domain that year. The year of each data point was considered as a variable (time trend) in each CART analysis performed to identify if variation in ceiling preemption occurred in specific years or time periods. We ran a separate CART analysis for each domain using all explanatory variables described above, except lung cancer deaths were only entered into the CART for tobacco control preemption, and firearm, homicide, and suicide deaths were only entered into CART for firearm safety preemption. We set the maximum tree depth to 5 and the minimum number of cases for parent and child nodes to 10 in order to generate shallower trees and facilitate ease of interpretation. We also specified a random seed to allow replication of analyses. Analyses were conducted using IBM SPSS Statistics 27 (IBM Corp, Armonk, NY) [35] and the SPSS Syntax is available in S2 File (SPSS Syntax).

## Results

**Fig 1:**
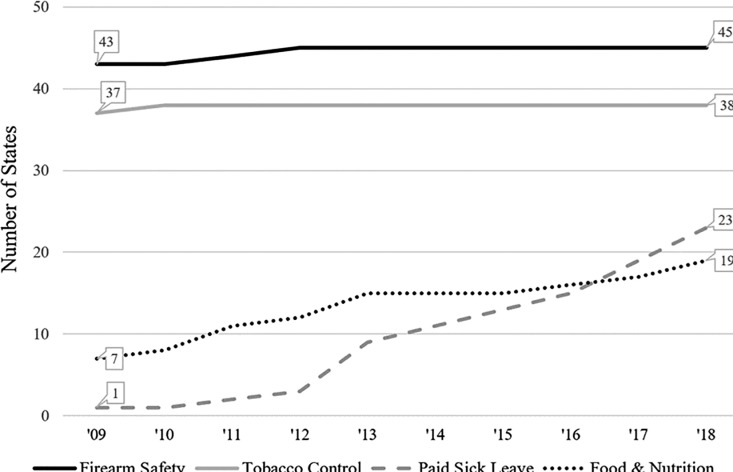
Number of States with Ceiling Preemption by Domain, 2009-2018.

As depicted in Fig 1, between 2009 and 2018 more states had ceiling preemption for tobacco control and firearms than for paid sick leave and food and nutrition policy. By 2018, 45 states had preemption laws for firearm safety, 38 states had preemption laws for tobacco control, 19 states had preemption laws for food and nutrition policy, and 23 states had preemption laws for paid sick leave. However, ceiling preemption also increased substantially between 2009 and 2018 in the food and nutrition policy and paid sick leave domains, whereas ceiling preemption increased minimally in the firearm safety and tobacco control domains. Specifically, states with ceiling preemption increased from one to 23 states for paid sick leave and from seven to 19 states for food and nutrition policy, whereas it only increased from 43 to 45 for firearm safety and from 37 to 38 for tobacco control.

Table 1 reports the number of domains preempted within each state in 2018 and the percentage of the U.S. population covered by state preemption laws in these domains. Only one state, New York, had no preemption in the four selected domains. Eight states had preemption laws in one domain, 15 states had laws in two domains, 17 states had laws in three domains, and nine states had laws covering all four domains. About 69 million people in the U.S. or 21% of the total population resided in states with state preemption laws in all four domains and an additional 154 million people (47%) lived in states with preemption in three domains.

**Table 1:** Number of domains preempted within each state, 2018.

States	Paid sick leave	Food & nutrition	Tobacco control	Firearm safety	Population covered^a^	
					Number of people	% U.S. population
**0 Domains** (1 State)					19,530,351	5.99
1 State *(NY)*					19,530,351	5.99
**1 Domain** (8 States)					21,524,188	6.60
3 States *(CT*, *HI*, *MA)*			♦		11,874,748	3.64
5 States *(AK*, *MN*, *NE*, *ND*, *VT)*				♦	9,649,440	2.96
**2 Domains** (15 States)					61,817,272	18.96
1 State *(RI)*	♦			♦	1,058,287	0.32
3 States *(AZ*, *ME*, *VA)*		♦		♦	16,998,367	5.21
11 States *(CO*, *ID*, *IL*, *MT*, *NV*, *NH*, *NM*, *PA*, *SD*, *WV*, *WY)*			♦	♦	43,760,618	13.42
**3 Domains** (17 States)					153,963,156	47.23
1 State *(NJ)*	♦	♦	♦		8,886,025	2.73
2 States *(GA*, *OH)*	♦	♦		♦	22,187,472	6.81
10 States *(AR*, *IN*, *IA*, *KY*, *LA*, *MD*, *MO*, *OK*, *SC*, *TX)*	♦		♦	♦	71,785,173	22.02
4 States (*CA*, *DE*, *UT*, *WA*)		♦	♦	♦	51,104,486	15.68
**4 Domains** (9 States)					69,150,987	21.21
9 States *(AL*, *FL*, *KS*, *MI*, *MS*, *NC*, *OR*, *TN*, *WI*)	♦	♦	♦	♦	69,150,987	21.21

^a^Population data from the 2009–2018 American Community Survey.

Table 2 reports the median and interquartile range of all explanatory variables considered by the CART algorithm and whether the variables were detected as a splitting variable in the classification tree for each domain. The trees for the food and nutrition policy and tobacco control domains were exclusively comprised of demographic splitting variables. The tree for the paid sick leave domain included three demographic variables and two health variables. Meanwhile the tree for the firearm safety domain included one demographic variable, one economic variable, and two health variables.

**Table 2:** Explanatory variables entered into CART and appearing in resulting Classification Trees, by Domain.

		Appears in classification tree			
Explanatory variables	Median (IQR)	Paid sick leave	Food & nutrition	Tobacco control	Firearm safety
**All Domains**					
**Demography**					
Population Size	4,476,401 (1,835,848–7,106,340)	♦	♦	♦	♦
% Under 18 Yrs. Old	23.13 (22.07–24.23)		♦		
% 18-64 Yrs. Old	62.22 (61.17–63.11)	♦			
% 65 Yrs. & Older	14.64 (13.55–15.87)		♦		
% NH AIAN	0.54 (0.29–1.20)		♦		
% NH API	2.85 (1.72–4.85)			♦	
% NH Black	7.93 (3.71–15.09)	♦	♦	♦	
% NH White	74.48 (60.39–82.61)			♦	
% Hispanic	8.70 (4.44–13.09)			♦	
**Economic Environment**					
Per Capita Income	44,107 (39,275–50,311)				
Median Income	58,680 (52,486–65,366)				
% in Poverty	13.90 (11.60–16.55)				
% Unemployed	6.00 (4.40–7.80)				♦
**Political Environment**					
Party Control					
Republican (N, %)	211 (42.2)				
Democrat (N, %)	112 (22.4)				
Split (N, %)	177 (35.4)				
% Voting (CPS Survey)^a^	55.70 (47.10–63.30)				
% Voting (Votes for Highest Office)^a^	53.30 (42.69–60.96)				
% Receiving SNAP	13.47 (10.56–16.02)				
% Receiving TANF	0.81 (0.47–1.15)				
% Uninsured	10.90 (8.10–14.30)				
**Health Outcomes & Behaviors**					
% Adult Obesity (*M, SD*)	29.06 (3.67)	♦			
% Adult Smokers	18.10 (15.90–21.00)				
% Flu Vaccine (*M, SD*)	45.68 (5.15)	♦			
**Other**					
YR	2013.5 (2.88)				
**Tobacco Preemption Only**					
Lung Cancer Deaths^a^ (*M, SD*)	43.52 (9.60)				
**Firearm Preemption Only**					
Firearm Deaths^a^	11.85 (9.0-15.0)				♦
Homicide Deaths^a^	5.20 (2.20-6.90)				
Suicide Deaths^a^	14.30 (12.30–17.80)				♦

IQR =  interquartile range, M = mean, SD =  standard deviation, NH =  non-Hispanic, AIAN =  American Indian and Alaska Native, API =  Asian or Pacific Islander, SNAP =  Supplemental Nutrition Assistance Program, TANF =  Temporary Assistance for Needy Families.

^a^Collected biannually.

^b^Age-adjusted deaths per 100,000.

The CART trees with the variable splits are presented in S1 Fig (Paid Sick Leave Preemption Classification Tree), S2 Fig (Food and Nutrition Preemption Classification Tree), S3 Fig (Tobacco Control Preemption Classification Tree), and S4 Fig (Firearm Safety Preemption Classification Tree). The tree for the paid sick leave domain first split on adult obesity followed by second-level splits on population size and percentage of NH Black, and third-, fourth-, and fifth-level splits on residents ages 18 to 64, population size, and the flu vaccination rate respectively. When split on obesity and NH Black residents, states with higher obesity prevalence and NH Black residents were more likely to have preemption. On the other hand, when split on residents ages 18 to 64 and flu vaccination, states with fewer working age adults and vaccination were more likely to have preemption. The relationship between population size and preemption varied depending on its location within the tree.

The tree for food and nutrition policy first split on NH Black residents, followed by second-level splits on residents under age 18 and again on NH Black residents. Third level splits occurred on residents ages 65 or older, residents under 18, and population size. Further splits occurred on NH Black residents and NH AIAN residents. When split on population size, states with fewer people were less likely to have preemption. When split on the percentage of residents ages 65 or over and NH AIAN residents, states with fewer older adults and states with more NH AIAN residents were more likely to have no preemption. The relationships between residents under age 18 and NH Black residents with preemption was dependent on the location of these variables within the tree.

The tree for the tobacco control domain first split on population size, followed by second-level splits again on population size and on NH white residents. A third-level split also occurred on population size again, a fourth-level split occurred on NH Black residents, and fifth-level splits occurred on Hispanic residents and NH API residents. When split on Hispanic and NH Black residents, states with more Hispanic residents and states with fewer NH Black residents were more likely to have preemption. When split on API and NH white residents, states with more of these residents were more likely to have no preemption. The relationship between population size and preemption was dependent on its location within the tree.

The tree for the firearm safety domain was the smallest of the four. The first split was on the number of firearm deaths, followed by second-level splits on suicide deaths and population size. The subgroup with higher firearm and suicide deaths also had a third-level split on the unemployment rate. When split on firearm and suicide deaths, states with more deaths are more likely to have firearm preemption. When split on population size, states with more people are more likely to have no preemption. When split on unemployment, states with a lower unemployment rate are slightly more likely to have preemption than states with a higher unemployment rate.

## Discussion

This study finds that specific population, demographic and economic characteristics in a state are associated with the adoption of ceiling preemption of paid sick, food and nutrition, tobacco, and firearm safety laws, but these characteristics vary by domain. Moreover, the relationship between specific factors and preemption in each of the four domains varied depending on the location of the factor within the trees. Our CART analysis showed that state demographics are a key factor in grouping states that had preemption laws from those that did not have these laws in all four domains. The total population appeared in all four trees, and the NH Black population appeared in the trees for paid sick leave, food and nutrition policy, and tobacco control. However, the precise relationship between population size and preemption varied depending on its location within the tree. For example, in states with a relatively high obesity prevalence (higher than 32.5%), the larger the total population size (higher than about 3 million people) the higher the likelihood of paid sick leave preemption. In states with an obesity rate lower than 32.5%, NH Black population of less than or equal to 30.6%, and a population 18 to 64 years of age of less than or equal to 62.1%, the larger the total population size (higher than about 4 million people) the higher the likelihood of paid sick preemption.

All the age- and race/ethnicity-related demographic variables appeared in at least one tree. Selected population health and economic indicators were also relevant. The prevalence of adult obesity and flu vaccination appeared in the paid sick leave tree and both firearm and suicide deaths appeared in the firearm safety preemption tree. The unemployment rate was relevant for preemption of firearm safety policy.

The CART analysis provides evidence of the association between state preemption and certain health outcomes directly relevant to the public health topic preempted. For example, the association between preempting firearm safety laws in a state and having higher firearm and suicide deaths is consistent with previous research positing that no preemption of firearm safety laws may be associated with lower firearm violence [22]. Further, paid sick leave preemption was associated with low flu vaccination rate; previous research found that having paid sick leave was associated with significantly higher odds of receiving a flu vaccine, and that rates of influenza may decrease substantially for workers with access to paid sick leave through local laws [5].

Our findings identified which populations within groups of states are affected by preemption. Across three domains, NH Black populations were associated with preemption. To the extent that ceiling preemption may be viewed as a coercive or punitive policy option for a state to pursue, this finding is consistent with findings of political scientists and sociologists who have observed that policy design and adoption may be affected by how population groups are affected, with those viewed negatively by policymakers likely to encounter far more punitive policies than those viewed positively [36–38]. Notably, a key finding in this study was that paid sick leave preemption was associated with large states with a high percentage of NH Black residents. There is a long history of states preempting local workers’ rights laws, especially in southern states with predominantly white legislatures and large NH Black populations [39–41]. Preemption in these contexts creates and exacerbates inequities while leaving local governments unable to enact policies that may reduce inequities and improve public health [39].

The policies targeted by preemption laws in this study are all ones that directly impact health disparities. Tobacco use (specifically cigarette smoking) disproportionately affects the health of people in low resource communities because they tend to smoke more heavily [42], have higher rates of lung cancer, and are diagnosed later in the disease process due to limited access to health care services [43]. Firearm violence disproportionately affects people and communities of color [44]. Paid sick leave allows workers to obtain medical care for themselves and their family with low-income workers [15] and racial and ethnic minorities [39] at the greatest risk of not having access to care due to lack of paid sick leave. Poor diets are associated with low socioeconomic attainment; even when certain populations report improvements in dietary habits, there have been persistent or worsening disparities based on race/ethnicity, education, and income [45,46]. Adopting evidence-based and innovative policies that can address the underlying causes of disease and disparities directly in these four domains has the potential to significantly improve the health of key demographic and socioeconomic groups; these are thus an appropriate area for policymaking by localities. Local governments are often in the best position to experiment with solutions to public health problems and are in a better position to address health disparities because they can identify and focus on communities at greatest risk, help develop community capacity, and coordinate community collaborations [47,48].

By identifying state-level characteristics that are associated with preemption, policymakers, public health practitioners, and advocates can anticipate which states may be more likely to enact preemptive laws in the future as a result of factors such as changes over time in population demographics. This information is useful to assess whether preemption laws considered or adopted in a given state also require considering or adopting protections for different population groups that could be adversely affected by these laws. Future research should also determine whether these findings can be extended to other countries with federalist systems.

Our study has several important limitations. First, our initial sourcing of the laws sought to identify all states and years with ceiling preemption in four domains, but some may not have been identified. Second, our classification system of preemption laws focused on the presence of ceiling preemption on any topic for each domain in a state and year; as such, we could not evaluate variations in preemption, the number of policies a state preempted for each domain, or whether a state had a preemption law prior to the year in question or kept the law after the study period. Third, we did not build a predictive model and evaluated its performance as we were interested in the state-level characteristics associated with preemption. Fourth, the measures considered for each state may not fully capture the complex factors, mechanisms, and system dynamics that lead to the adoption of preemption laws. Fifth, this study did not include consideration of states enacting substantive laws to fill the regulatory void; however, because the goal of this study was to examine preemption, substantive lawmaking would not have changed the results. Nonetheless, previous research indicates that few states enact substantive policies alongside preemption [4,5,7].

## Conclusions

State preemption laws are often used to remove local policymaking authority in areas relevant to public health. Population make-up, economic characteristics, and health measures in a state were associated with preemption laws across states. Changes over time in these indicators can provide useful information to policymakers, public health practitioners, and advocates interested in knowing whether preemption laws could find fertile ground in a given state and policy domain relevant to public health.

## Supporting information

S1 Fig Paid sick leave preemption classification tree. “Yes” =  the presence of ceiling preemption of local paid sick leave policies, NH =  Non-Hispanic, Pop. = Population, Yrs. =  years.
(TIF)

S2 Fig Food and nutrition preemption classification tree. “Yes” =  the presence of ceiling preemption of local food and nutrition policies, NH = Non-Hispanic, AIAN =  American Indian/Alaska Native, Pop. = Population, Yrs. =  years.
(TIF)

S3 Fig Tobacco Control Preemption Classification Tree. “Yes” =  the presence of ceiling preemption of local tobacco control policies, NH =  Non-Hispanic, Hisp =  Hispanic, API =  Asian/Pacific Islander, Pop. = Population.
(TIF)

S4 Fig Firearm safety preemption classification tree. “Yes” =  the presence of ceiling preemption of local firearm safety policies, Pop. =  Population, Firearm deaths =  age-adjusted firearm-related deaths per 100,000 people, Suicide deaths =  age-adjusted deaths due to suicide per 100,000 people.
(TIF)

S1 File CART input data file and variable descriptions.
(XLSX)

S2 File SPSS syntax.
(PDF)
