# Supplementary figures and images for: Population, demographic and socioeconomic characteristics associated with state preemption laws in the United States, 2009–2018
Source: PLoS One. 2025 Apr 4;20(4):e0321184. doi: 10.1371/journal.pone.0321184 (PMC11970670; doi:10.1371/journal.pone.0321184)

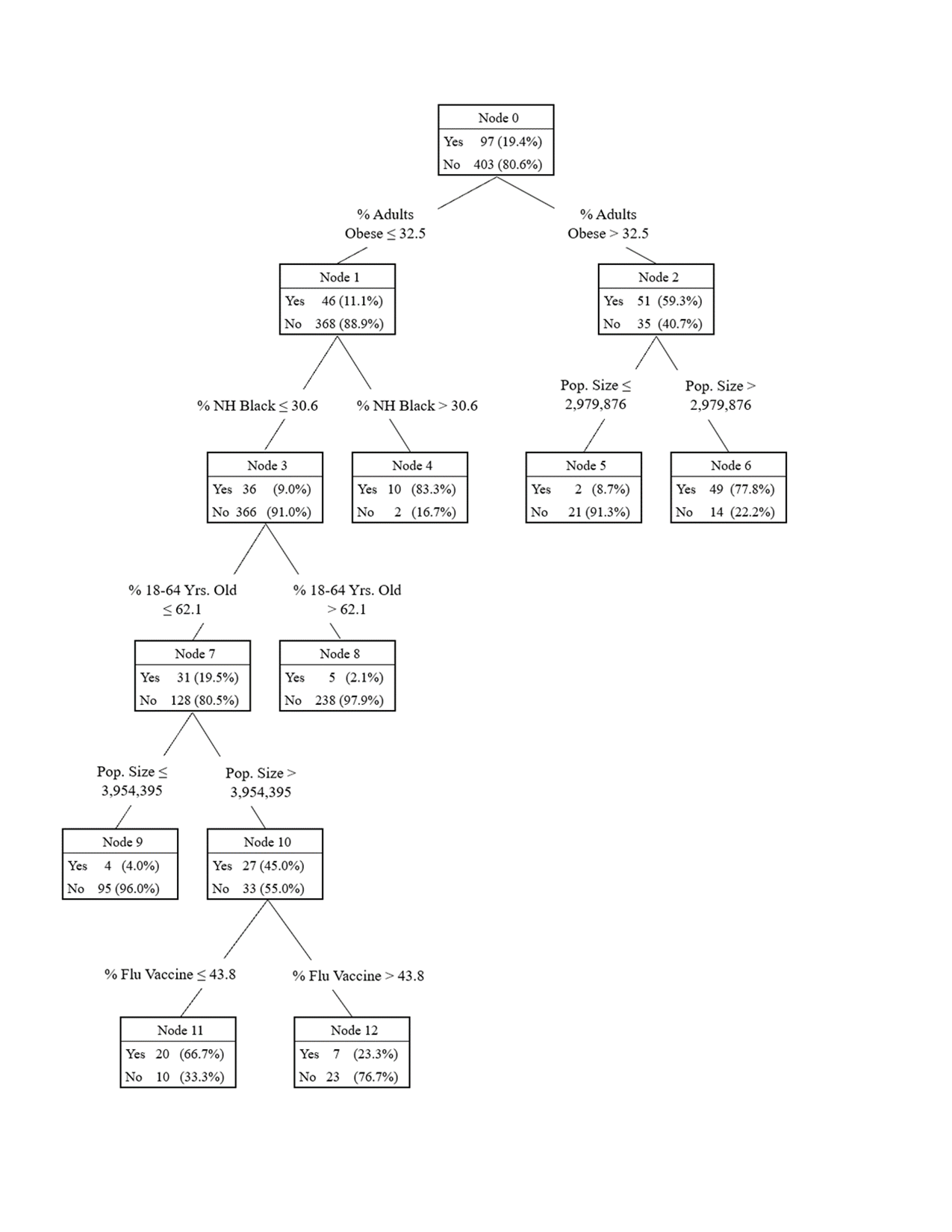

Supplement: S1 Fig — “Yes” = the presence of ceiling preemption of local paid sick leave policies, NH = Non-Hispanic, Pop. = Population, Yrs. = years. (TIF) [file pone.0321184.s001.tif]

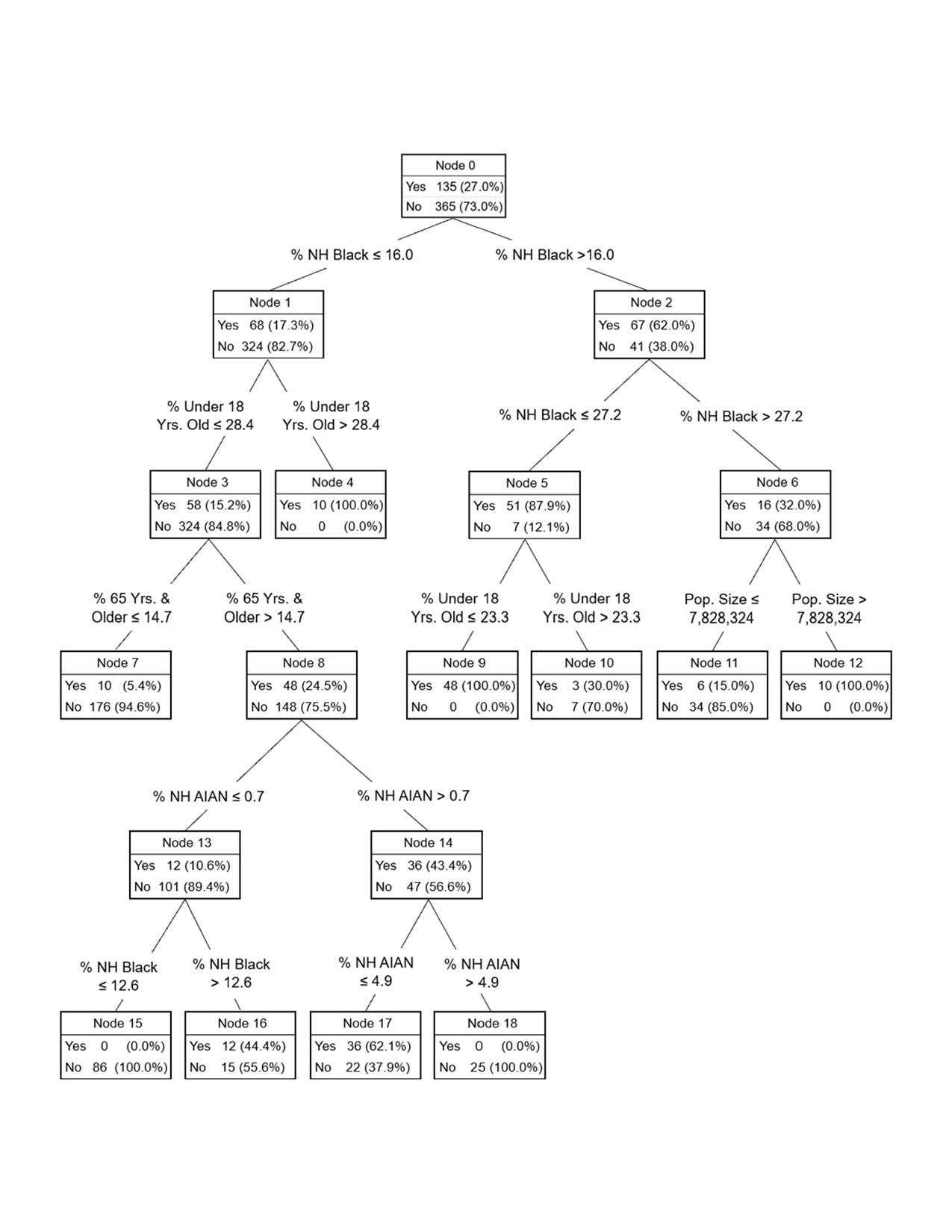

Supplement: S2 Fig — “Yes” = the presence of ceiling preemption of local food and nutrition policies, NH = Non-Hispanic, AIAN = American Indian/Alaska Native, Pop. = Population, Yrs. = years. (TIF) [file pone.0321184.s002.tif]

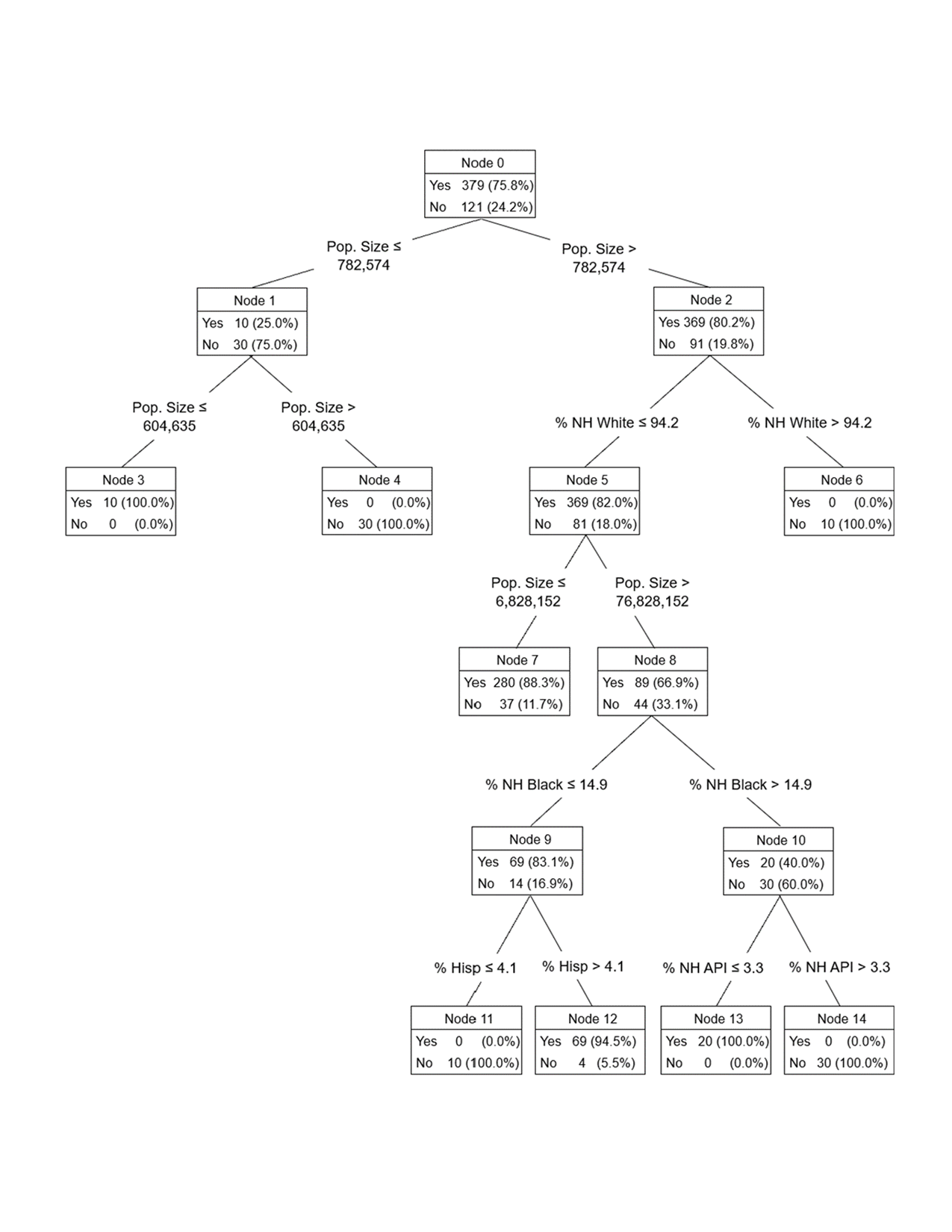

Supplement: S3 Fig — “Yes” = the presence of ceiling preemption of local tobacco control policies, NH = Non-Hispanic, Hisp = Hispanic, API = Asian/Pacific Islander, Pop. = Population. (TIF) [file pone.0321184.s003.tif]

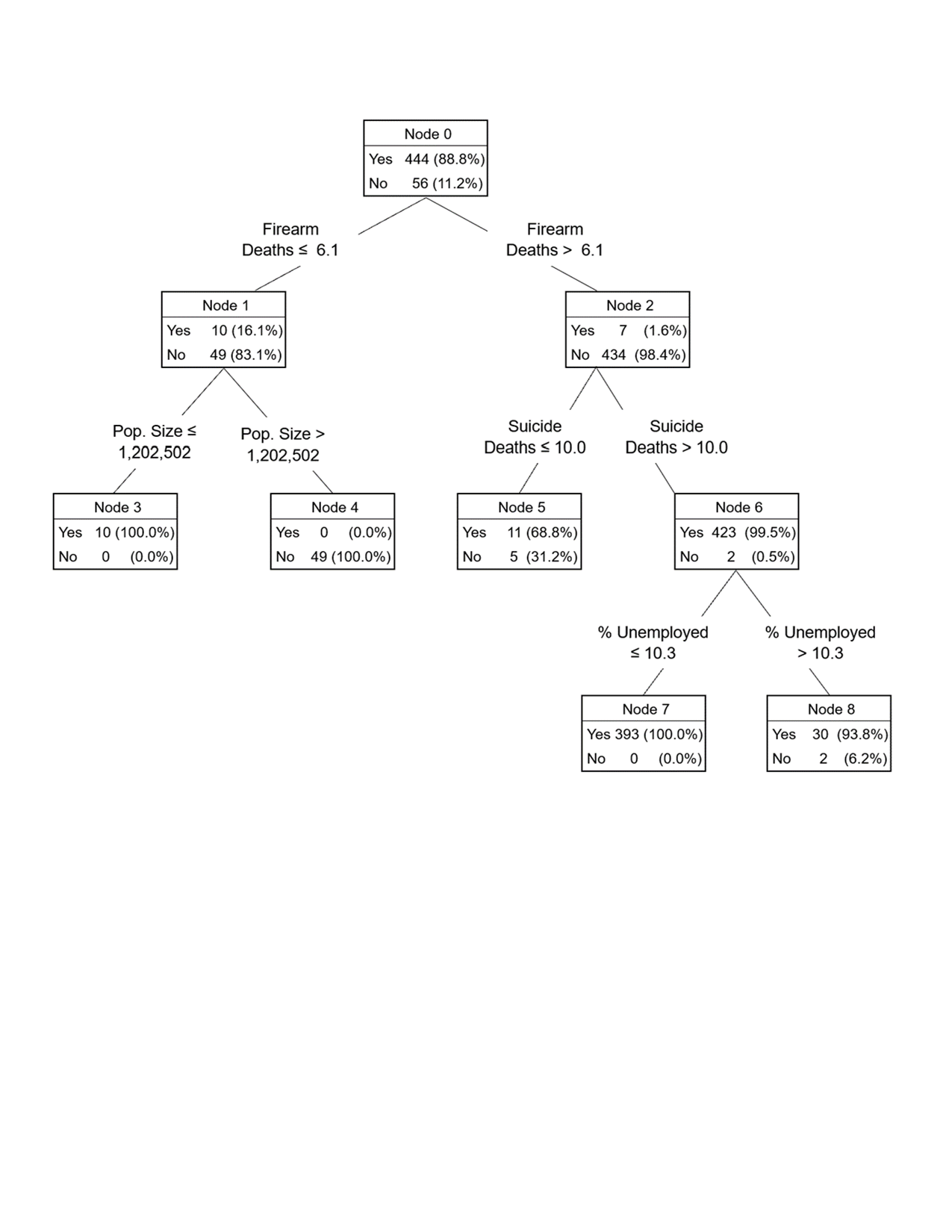

Supplement: S4 Fig — “Yes” = the presence of ceiling preemption of local firearm safety policies, Pop. = Population, Firearm deaths = age-adjusted firearm-related deaths per 100,000 people, Suicide deaths = age-adjusted deaths due to suicide per 100,000 people. (TIF) [file pone.0321184.s004.tif]
