# Supplementary material for: Population, demographic and socioeconomic characteristics associated with state preemption laws in the United States, 2009–2018
Source: PLoS One. 2025 Apr 4;20(4):e0321184. doi: 10.1371/journal.pone.0321184 (PMC11970670; doi:10.1371/journal.pone.0321184)
Supplement: S2 File — (PDF) [file pone.0321184.s006.pdf]

Cart Analysis: SPSS Syntax

```
TREE SickPre [n] BY YR [s] PopSize [s] PercUnder18 [s] Perc18to64 [s] Perc65plus [s] PercAIAN [s]
    PercAPI [s] PercBlack [s] PercWhite[s] PercHisp [s] PerCapInc [s] MedInc [s] PercPov [s] PercUnemp [s]
    RepControl [n] DemControl [n] SplitControl [n] Vote_HighOff [s] Vote_CPS [s] PercUninsure [s] PercSNAP [s]
    PercTANF [s] PercObese [s] PercSmoke [s] PercFlu [s] Sick_Law [n]
  /TREE DISPLAY=TOPDOWN NODES=STATISTICS BRANCHSTATISTICS=YES NODEDEFS=YES
SCALE=AUTO
  /DEPCATEGORIES USEVALUES=[VALID]
  /PRINT MODELSUMMARY IMPORTANCE SURROGATES CLASSIFICATION RISK TREETABLE
  /SAVE NODEID PREDVAL
  /METHOD TYPE=CRT MAXSURROGATES=AUTO PRUNE=NONE
  /GROWTHLIMIT MAXDEPTH=AUTO MINPARENTSIZE=10 MINCHILDSIZE=10
  /VALIDATION TYPE=NONE OUTPUT=BOTHSAMPLES
  /CRT IMPURITY=GINI MINIMPROVEMENT=0.0001
  /COSTS EQUAL
  /PRIORS FROMDATA ADJUST=NO
 /MISSING NOMINALMISSING=MISSING.


TREE FoodPre [n] BY YR [s] PopSize [s] PercUnder18 [s] Perc18to64 [s] Perc65plus [s] PercAIAN [s] PercAPI [s]
    PercBlack [s] PercWhite [s] PercHisp [s] PerCapInc [s] MedInc [s] PercPov [s] PercUnemp [s] RepControl [n]
    DemControl [n] SplitControl [n] Vote_HighOff [s] Vote_CPS [s] PercUninsure [s] PercSNAP [s]
    PercTANF [s] PercObese [s] PercSmoke [s] PercFlu [s]
  /TREE DISPLAY=TOPDOWN NODES=STATISTICS BRANCHSTATISTICS=YES NODEDEFS=YES
SCALE=AUTO
  /DEPCATEGORIES USEVALUES=[VALID]
  /PRINT MODELSUMMARY IMPORTANCE SURROGATES CLASSIFICATION RISK TREETABLE
  /SAVE NODEID PREDVAL
  /METHOD TYPE=CRT MAXSURROGATES=AUTO PRUNE=NONE
  /GROWTHLIMIT MAXDEPTH=AUTO MINPARENTSIZE=10 MINCHILDSIZE=10
  /VALIDATION TYPE=NONE OUTPUT=BOTHSAMPLES
  /CRT IMPURITY=GINI MINIMPROVEMENT=0.0001

TREE TobPre [n] BY YR [s] PopSize [s] PercUnder18 [s] Perc18to64 [s] Perc65plus [s] PercAIAN [s] PercAPI [s]
    PercBlack [s] PercWhite [s] PercHisp [s] PerCapInc [s] MedInc [s] PercPov [s] PercUnemp [s] RepControl [n]
    DemControl [n] SplitControl [n] Vote_HighOff [s] Vote_CPS [s] PercUninsure [s] PercSNAP [s]
    PercTANF [s] PercObese [s] PercSmoke [s] PercFlu [s] LungCancer [s]
  /TREE DISPLAY=TOPDOWN NODES=STATISTICS BRANCHSTATISTICS=YES NODEDEFS=YES
SCALE=AUTO
  /DEPCATEGORIES USEVALUES=[VALID]
```

```
  /PRINT MODELSUMMARY IMPORTANCE SURROGATES CLASSIFICATION RISK TREETABLE
  /SAVE NODEID PREDVAL
  /METHOD TYPE=CRT MAXSURROGATES=AUTO PRUNE=NONE
  /GROWTHLIMIT MAXDEPTH=AUTO MINPARENTSIZE=10 MINCHILDSIZE=10
  /VALIDATION TYPE=NONE OUTPUT=BOTHSAMPLES
  /CRT IMPURITY=GINI MINIMPROVEMENT=0.0001
  /COSTS EQUAL
  /PRIORS FROMDATA ADJUST=NO
 /MISSING NOMINALMISSING=MISSING.

TREE FirearmPre [n] BY YR [s] PopSize [s] PercUnder18 [s] Perc18to64 [s] Perc65plus [s] PercAIAN [s]
    PercAPI [s] PercBlack [s] PercWhite [s] PercHisp [s] PerCapInc [s] MedInc [s] PercPov [s]
    PercUnemp [s] RepControl [n] DemControl [n] SplitControl [n] Vote_HighOff [s] Vote_CPS [s] PercUninsure [s]
    PercSNAP [s] PercTANF [s] PercObese [s] PercSmoke [s] PercFlu [s] Firearm [s] Homicide [s] Suicide [s]
  /TREE DISPLAY=TOPDOWN NODES=STATISTICS BRANCHSTATISTICS=YES NODEDEFS=YES
SCALE=AUTO
  /DEPCATEGORIES USEVALUES=[VALID]
  /PRINT MODELSUMMARY IMPORTANCE SURROGATES CLASSIFICATION RISK TREETABLE
  /SAVE NODEID PREDVAL
  /METHOD TYPE=CRT MAXSURROGATES=AUTO PRUNE=NONE
  /GROWTHLIMIT MAXDEPTH=AUTO MINPARENTSIZE=10 MINCHILDSIZE=10
  /VALIDATION TYPE=NONE OUTPUT=BOTHSAMPLES
  /CRT IMPURITY=GINI MINIMPROVEMENT=0.0001
  /COSTS EQUAL
  /PRIORS FROMDATA ADJUST=NO
 /MISSING NOMINALMISSING=MISSING.
```
